# Incidence, Predictors, and Clinical Outcomes of Acute Kidney Injury in Critically Ill Patients: A Retrospective Study

**DOI:** 10.7759/cureus.117498

**Published:** 2026-10-05

**Authors:** Aisha Butt, Arman Amjad, Amna Rehman, Ali Raza, FNU Hamna, Hashir Rana, Akbar Bashir

**Affiliations:** 1 General Surgery, Services Hospital, Lahore, PAK; 2 Emergency Medicine, Maidstone and Tunbridge Wells NHS Trust, Tunbridge Wells, GBR; 3 Internal Medicine, Islamabad Medical and Dental College, Islamabad, PAK; 4 Emergency Medicine, Shalamar Hospital, Lahore, PAK; 5 Internal Medicine, Sir Ganga Ram Hospital, Lahore, PAK; 6 Acute Medicine, Northampton General Hospital, Northampton, GBR; 7 Internal Medicine, Services Hospital, Lahore, PAK

**Keywords:** acute kidney injury, critical illness, intensive care units, mortality, renal replacement therapy, sepsis

## Abstract

Background

Acute kidney injury (AKI) is a frequent and serious complication among critically ill patients and is associated with prolonged hospitalization, increased need for organ support, and higher mortality.

Objective

The objective of the study is to determine the incidence of AKI among critically ill patients, identify its independent predictors, and evaluate its association with major clinical outcomes.

Methods

This retrospective observational study was conducted at Services Hospital, Lahore, Pakistan, from June 2023 to June 2026 and included 261 adult critically ill patients. Demographic characteristics, comorbidities, clinical severity scores, laboratory parameters, treatment-related factors, and clinical outcomes were extracted from medical records.

Results

AKI developed in 102 of 261 patients, corresponding to an incidence of 39.1%. Among patients with AKI, 42 (41.2%) had Kidney Disease: Improving Global Outcomes (KDIGO) stage 1, 34 (33.3%) had stage 2, and 26 (25.5%) had stage 3 disease. Compared with patients without AKI, those with AKI had higher intensive care unit (ICU) mortality (35 (34.3%) vs. 19 (11.9%)), in-hospital mortality (41 (40.2%) vs. 24 (15.1%)), and renal replacement therapy requirement (21 (20.6%) vs. 0 (0.0%)), as well as longer ICU and hospital stays and longer duration of mechanical ventilation (all p < 0.001). Factors independently associated with AKI included chronic kidney disease (adjusted odds ratio (aOR) 3.12, 95% confidence interval (CI) 1.34-7.28), vasopressor support at ICU admission (aOR 2.86, 95% CI 1.49-5.50), sepsis or septic shock (aOR 2.74, 95% CI 1.48-5.08), mechanical ventilation at ICU admission (aOR 2.09, 95% CI 1.08-4.04), increasing serum lactate (aOR 1.31, 95% CI 1.10-1.57), higher Acute Physiology and Chronic Health Evaluation II (APACHE II) score (aOR 1.09, 95% CI 1.04-1.14), and increasing age (aOR 1.03, 95% CI 1.01-1.05).

Conclusion

AKI affected more than one-third of critically ill patients and was associated with a greater observed clinical burden, including higher mortality, greater need for renal replacement therapy, prolonged mechanical ventilation, and longer ICU and hospital stays. Chronic kidney disease, sepsis or septic shock, vasopressor support at ICU admission, mechanical ventilation at ICU admission, higher serum lactate, higher APACHE II score, and increasing age were independently associated with AKI. Because clinical outcome comparisons were unadjusted, the observed differences in mortality, mechanical ventilation duration, and length of stay do not establish AKI as an independent determinant of these outcomes.

## Introduction

Acute kidney injury (AKI) is a common and severe condition that occurs in the intensive care unit (ICU) setting in critically ill patients. It is defined as a sudden drop in renal function with reduced elimination of metabolic waste products and fluid, electrolyte, and acid-base disturbances [1]. Although critical care medicine has come a long way, AKI continues to be related to significant morbidity and mortality, longer duration in the ICU and hospital, and higher healthcare expenses [2]. The frequency of AKI in critically ill patients is reported to be between 30% and 60%, and it is associated with different proportions of renal replacement therapy (RRT) use, depending on the clinical setting, patient characteristics, and the definition of AKI [3].

The etiology of AKI in critically ill patients is typically complex. Some clinical factors associated with the onset of AKI are important, including sepsis, septic shock, hypovolemia, major surgery and trauma, nephrotoxic drugs or iodinated contrast, mechanical ventilation, and underlying chronic kidney disease (CKD) [4]. These factors can be single or multiple and may lead to renal injury via renal hypoperfusion, renal inflammation, endothelial and microcirculatory dysfunction, oxidative stress, and direct tubular damage [5].

Thus, the early identification of AKI is of great importance as it is associated with adverse clinical outcomes, even with mild decrements in kidney function. The widely used Kidney Disease: Improving Global Outcomes (KDIGO) criteria for the diagnosis and staging of AKI are based on changes in serum creatinine and urine output [6]. There is a correlation between the severity of AKI and longer length of stay (LOS) in the ICU and hospital, higher need for mechanical ventilation and vasopressor use, higher rate of requiring RRT, more progression to CKD, and higher short- and long-term mortality rates [7]. Other demographic, clinical, and laboratory parameters have also been linked to the onset of AKI in critically ill patients [8]. The factors reported to be associated with risk are advanced age, diabetes mellitus, hypertension, preexisting CKD, prolonged hypotension, requirement for vasopressors, elevated serum lactate, mechanical ventilation, and exposure to nephrotoxic agents [9].

Neutrophil gelatinase-associated lipocalin (NGAL), cystatin C, and kidney injury molecule-1 (KIM-1) are several biomarkers that have been studied as potential early markers of kidney injury. Their use is, however, limited in many clinical settings, due to cost, availability, and laboratory requirements [10]. Thus, serum creatinine and urine output continue to be the major parameters relied upon in the diagnosis and staging of AKI in everyday clinical practice. Reported incidence, risk factors, and outcomes of AKI differ significantly between studies and are linked to patient characteristics, case mix of the ICU, resources, and clinical practice [11].

Data regarding AKI in critically ill patients in low- and middle-income countries (LMICs) are still relatively scarce. The incidence and impact of AKI in these environments can be affected by delayed presentation, high burden of severe AKI, limited access to RRT, and limited healthcare resources [12]. Therefore, locally generated evidence is crucial in identifying patients who are at high risk of developing AKI to guide preventive and monitoring measures and enhance critical care outcomes [13].

Objective

The objective of the study is to ascertain the prevalence of AKI in the critically ill, factors that may be independently associated with AKI, and its association with key clinical outcomes.

## Materials and methods

Study design and setting

It was a retrospective observational study carried out at Services Hospital, Lahore, Pakistan, from June 2023 to 2026. A total of 261 adult patients (aged 18 years and older) with predefined inclusion criteria were included in the analysis during the study period. Consecutive sampling (non-probability sampling technique) was used. Eligible patients were selected from the electronic medical record (EMR) system of the hospital, and consecutive patients who met the inclusion criteria between enrollment and the completion of the predetermined sample during the study period were included.

Inclusion and exclusion criteria

Inclusion criteria were age ≥ 18 years and admission to the ICU for at least 24 hours with a complete clinical and laboratory record. The following patients were excluded: end-stage renal disease on maintenance dialysis, prior kidney transplantation, chronic dialysis prior to admission to the ICU, incomplete records, and ICU stay < 24 hours.

Data collection

Data were retrospectively extracted from EMRs using a structured data collection form developed by the authors specifically for the present study. Demographic variables were age, sex, body mass index (BMI), smoking status, and related comorbidities such as diabetes mellitus, hypertension, CKD, chronic liver disease (CLD), ischemic heart disease, and malignancy. The complete data collection form is provided in the appendix.

The clinical parameters collected at admission to the ICU were the primary diagnosis, the need for vasopressor support, mechanical ventilation, Acute Physiology and Chronic Health Evaluation II (APACHE II) score [14], Sequential Organ Failure Assessment (SOFA) score [15], and mean arterial pressure (MAP). Laboratory parameters at admission to the ICU were serum creatinine, blood urea nitrogen (BUN), serum lactate, hemoglobin, white blood cell (WBC) count, serum albumin, electrolyte profile, and arterial blood gas parameters. The pre-AKI clinical and laboratory characteristics were measured prior to AKI onset and subsequently analyzed as potential AKI predictors.

The urine output was measured during the entire period of the hospital stay for the assessment of AKI by the KDIGO criteria [7]. Potentially nephrotoxic drugs, iodinated contrast, surgery, and transfusion were also documented. Exposures that occurred prior to development of AKI were considered to be potential predictors, while exposures that occurred after the onset of AKI were counted as subsequent clinical exposures but not considered to be potential predictors.

Diagnosis and staging of AKI were made based on the KDIGO criteria with a change in serum creatinine and urine output. The patients were then divided into two groups: patients who developed AKI and patients who did not develop AKI during their stay in the ICU.

Clinical outcomes measured were the need for RRT, mechanical ventilation, LOS in the ICU and hospital, and mortality in the ICU and in the hospital. The main outcome was the occurrence of AKI in patients admitted to the ICU during their critical illness. Secondary outcomes included independent risk factors for subsequent AKI, as well as clinical outcomes related to AKI such as requirement for RRT, mechanical ventilation duration, index ICU and hospital mortality, and index ICU or in-hospital LOS.

AKI ascertainment

AKI was identified and staged according to the KDIGO criteria, based on changes in serum creatinine and urine output. For serum creatinine-based assessment, the baseline value was defined as the most recent documented serum creatinine available before or at ICU admission, provided that it represented the patient's pre-AKI renal function. When a preadmission creatinine value was unavailable, the earliest serum creatinine measured at ICU admission was used as the reference value for subsequent changes during the ICU stay. No estimated baseline creatinine was imputed when a documented value was unavailable.

AKI was defined as any of the following: an increase in serum creatinine by ≥0.3 mg/dL within 48 hours, an increase in serum creatinine to ≥1.5 times the reference value occurring within the preceding seven days, or urine output < 0.5 mL/kg/hour for ≥6 hours. KDIGO stage 1 was defined as a serum creatinine increase of ≥0.3 mg/dL within 48 hours or 1.5-1.9 times the reference value, or urine output < 0.5 mL/kg/hour for 6-12 hours. Stage 2 was defined as a serum creatinine increase to 2.0-2.9 times the reference value or urine output < 0.5 mL/kg/hour for ≥12 hours. Stage 3 was defined as a serum creatinine increase to ≥3.0 times the reference value, an increase to ≥4.0 mg/dL, initiation of RRT, or urine output < 0.3 mL/kg/hour for ≥24 hours or anuria for ≥12 hours.

Urine output was abstracted from the ICU fluid-balance and nursing records and assessed according to the documented hourly urine output and patient body weight. When urine output documentation was incomplete or insufficient to establish a KDIGO urine output criterion, AKI classification was based on the available serum creatinine criteria rather than assigning an AKI stage solely from incomplete urine output data. The date and time of the first criterion fulfilling the KDIGO definition were recorded as the AKI onset time. AKI severity was classified according to the highest KDIGO stage reached during the ICU admission.

Statistical analysis

Analytical statistics in IBM SPSS Statistics version 27.0 (IBM Corp., Armonk, NY, USA) was used for statistical analysis. The distribution of continuous variables was assessed using the Shapiro-Wilk test. Normally distributed continuous variables were expressed as mean ± standard deviation (SD) and compared between the AKI and non-AKI groups using the independent-samples t-test. The Mann-Whitney U test was used to compare the median with the interquartile range (IQR) of non-normally distributed continuous variables. The chi-squared test or Fisher's exact test, depending on the circumstance, was used to compare the frequencies and percentages of the categorical variables. A two-sided p-value < 0.05 was considered statistically significant.

A multivariable binary logistic regression model was constructed to identify factors independently associated with subsequent development of AKI. Candidate variables were selected a priori based on clinical relevance and supported by the existing literature, together with variables showing an association with AKI on univariable analysis. Candidate variables considered included age, sex, diabetes mellitus, hypertension, CKD, ischemic heart disease, CLD, malignancy, sepsis/septic shock, MAP, mechanical ventilation at ICU admission, vasopressor requirement at ICU admission, admission serum creatinine, BUN, serum lactate, serum albumin, hemoglobin, WBC count, and other clinically relevant admission characteristics. Variables occurring after AKI onset were not considered candidate predictors.

For variables selected on the basis of univariable analysis, a prespecified p-value threshold of <0.20 was used for consideration in the multivariable model, while clinically important variables were retained irrespective of their univariable p-value. Age, APACHE II score, and serum lactate were entered as continuous variables rather than categorized.

Potential multicollinearity among candidate predictors was assessed before fitting the final model using variance inflation factors and tolerance statistics. Variables representing closely related measures of illness severity, including APACHE II score, SOFA score, sepsis/septic shock, vasopressor requirement, mechanical ventilation, and serum lactate, were examined for collinearity before final model specification.

Model performance was evaluated using the Hosmer-Lemeshow goodness-of-fit test for calibration and the area under the receiver operating characteristic curve (AUC) for discrimination. Adjusted odds ratios (aORs), 95% confidence intervals (CIs), and two-sided p-values were reported. Statistical significance was defined as p < 0.05.

## Results

A total of 261 critically ill patients were included in the analysis. The mean age was 58.7 ± 15.2 years, and 158 (60.5%) patients were male. Patients who developed AKI were older than those who did not develop AKI (63.4 ± 13.8 vs. 55.7 ± 15.4 years). Diabetes mellitus, hypertension, and CKD were more frequent among patients who developed AKI, occurring in 46 (45.1%), 55 (53.9%), and 23 (22.5%) patients, respectively, compared with 43 (27.0%), 63 (39.6%), and 12 (7.5%) patients in the non-AKI group. Sepsis or septic shock was present in 62 (60.8%) patients with AKI compared with 54 (34.0%) patients without AKI. APACHE II and SOFA scores were also higher among patients who developed AKI (Table 1).

**Table 1:** Baseline demographic and clinical characteristics of critically ill patients according to AKI status (n = 261) Values are presented as mean ± standard deviation (SD) or number (percentage), as appropriate. AKI: acute kidney injury; BMI: body mass index; APACHE II [14]: Acute Physiology and Chronic Health Evaluation II; SOFA [15]: Sequential Organ Failure Assessment

Variable	Total (n = 261)	AKI (n = 102)	No AKI (n = 159)
Age (years)	58.7 ± 15.2	63.4 ± 13.8	55.7 ± 15.4
Male sex	158 (60.5)	65 (63.7)	93 (58.5)
BMI (kg/m²)	26.8 ± 4.9	27.3 ± 5.1	26.5 ± 4.7
Diabetes mellitus	89 (34.1)	46 (45.1)	43 (27.0)
Hypertension	118 (45.2)	55 (53.9)	63 (39.6)
Chronic kidney disease	35 (13.4)	23 (22.5)	12 (7.5)
Ischemic heart disease	52 (19.9)	25 (24.5)	27 (17.0)
Chronic liver disease	24 (9.2)	12 (11.8)	12 (7.5)
Malignancy	31 (11.9)	14 (13.7)	17 (10.7)
Sepsis/septic shock	116 (44.4)	62 (60.8)	54 (34.0)
APACHE II score [14]	18.9 ± 7.2	23.1 ± 6.8	16.2 ± 6.1
SOFA score [15]	7.1 ± 3.4	9.2 ± 3.3	5.8 ± 2.8

AKI developed in 102 of 261 patients, corresponding to an incidence of 39.1%, while 159 (60.9%) patients did not develop AKI. Among patients who developed AKI, KDIGO stage 1 was the most frequent category, occurring in 42 (41.2%) patients, followed by stage 2 in 34 (33.3%) and stage 3 in 26 (25.5%) patients. Expressed as proportions of the total study population, KDIGO stages 1, 2, and 3 accounted for 42 (16.1%), 34 (13.0%), and 26 (10.0%), respectively (Table 2).

**Table 2:** Incidence and severity of AKI according to KDIGO classification (n = 261) Values are presented as number (percentage). Percentages for individual KDIGO stages are calculated among patients who developed AKI (n = 102), whereas percentages for the overall cohort are calculated using the total study population (n = 261). AKI: acute kidney injury; KDIGO [7]: Kidney Disease: Improving Global Outcomes

AKI classification	n (%) of total sample	n (%) among AKI patients
No AKI	159 (60.9)	-
Any AKI	102 (39.1)	102 (100.0)
KDIGO [7] stage 1	42 (16.1)	42 (41.2)
KDIGO[7] stage 2	34 (13.0)	34 (33.3)
KDIGO[7] stage 3	26 (10.0)	26 (25.5)
Renal replacement therapy required	21 (8.0)	21 (20.6)

RRT was required in 21 (20.6%) patients with AKI, corresponding to 21 (8.0%) of the total study population. No patient without AKI required RRT (Table 2).

Serum creatinine at ICU admission, BUN, serum lactate, and WBC count were significantly higher among patients who subsequently developed AKI, whereas serum albumin, hemoglobin, and MAP were significantly lower than in patients who did not develop AKI (Table 3).

**Table 3:** Laboratory and treatment-related characteristics according to AKI status Values are presented as mean ± standard deviation (SD) or number (percentage), as appropriate. Continuous variables were compared using the independent-samples t-test, and categorical variables were compared using the chi-squared test or Fisher’s exact test, as appropriate. Mechanical ventilation and vasopressor support refer to status at ICU admission. Nephrotoxic drug exposure, iodinated contrast exposure, and blood transfusion represent exposures documented during the ICU stay and are presented descriptively according to AKI status; their temporal relationship to AKI onset was not assumed. AKI: acute kidney injury; ICU: intensive care unit

Variable	AKI (n = 102)	No AKI (n = 159)	p-value
Serum creatinine at ICU admission (mg/dL)	1.31 ± 0.58	0.89 ± 0.31	<0.001
Blood urea nitrogen (mg/dL)	41.8 ± 18.7	27.4 ± 12.6	<0.001
Serum lactate (mmol/L)	4.3 ± 2.1	2.5 ± 1.4	<0.001
Serum albumin (g/dL)	2.84 ± 0.61	3.29 ± 0.58	<0.001
Hemoglobin (g/dL)	10.4 ± 2.1	11.2 ± 2.0	0.002
White blood cell count (×10⁹/L)	14.8 ± 6.7	11.9 ± 5.3	<0.001
Mean arterial pressure (mmHg)	68.9 ± 11.5	77.6 ± 12.3	<0.001
Mechanical ventilation	78 (76.5)	76 (47.8)	<0.001
Vasopressor support	71 (69.6)	57 (35.8)	<0.001
Nephrotoxic drug exposure	44 (43.1)	39 (24.5)	0.002
Iodinated contrast exposure	31 (30.4)	30 (18.9)	0.032
Blood transfusion	37 (36.3)	32 (20.1)	0.004

Mechanical ventilation at ICU admission was present in 78 (76.5%) patients who subsequently developed AKI compared with 76 (47.8%) patients without AKI (p < 0.001). Similarly, vasopressor support at ICU admission was more frequent among patients who developed AKI than among those who did not (71 (69.6%) vs. 57 (35.8%), p < 0.001).

Nephrotoxic drug exposure was documented during the ICU stay in 44 (43.1%) patients with AKI compared with 39 (24.5%) patients without AKI (p = 0.002). Iodinated contrast exposure and blood transfusion were also more frequently documented among patients with AKI than among those without AKI (31 (30.4%) vs. 30 (18.9%), p = 0.032, and 37 (36.3%) vs. 32 (20.1%), p = 0.004, respectively) (Table 3).

Patients who developed AKI had a longer median duration of mechanical ventilation than those without AKI (7 (4-12) vs. 4 (2-7) days, p < 0.001). The median ICU LOS was also longer among patients with AKI (10 (7-16) vs. 6 (4-10) days, p < 0.001), as was the median hospital LOS (18 (12-27) vs. 12 (8-19) days, p < 0.001).

ICU mortality was significantly higher among patients with AKI than among those without AKI (35 (34.3%) vs. 19 (11.9%), p < 0.001). Similarly, in-hospital mortality was higher in the AKI group (41 (40.2%) vs. 24 (15.1%), p < 0.001). Overall, 61 (59.8%) patients with AKI and 135 (84.9%) patients without AKI were discharged alive (p < 0.001). RRT was required in 21 (20.6%) patients with AKI compared with none of the patients without AKI (p < 0.001) (Table 4).

**Table 4:** Clinical outcomes according to AKI status Values are presented as median (IQR) for continuous variables and number (percentage) for categorical variables. Continuous variables were compared using the Mann-Whitney U test, and categorical variables were compared using the chi-squared test or Fisher’s exact test, as appropriate. AKI: acute kidney injury; ICU: intensive care unit; IQR: interquartile range

Outcome	AKI (n = 102)	No AKI (n = 159)	p-value
Renal replacement therapy	21 (20.6)	0 (0.0)	<0.001
Mechanical ventilation duration (days)	7 (4-12)	4 (2-7)	<0.001
ICU length of stay (days)	10 (7-16)	6 (4-10)	<0.001
Hospital length of stay (days)	18 (12-27)	12 (8-19)	<0.001
ICU mortality	35 (34.3)	19 (11.9)	<0.001
In-hospital mortality	41 (40.2)	24 (15.1)	<0.001
Discharged alive	61 (59.8)	135 (84.9)	<0.001

Multivariable binary logistic regression analysis identified several factors independently associated with subsequent AKI. Increasing age was independently associated with higher odds of AKI, with a 3% increase in the odds of AKI for each additional year of age (aOR 1.03, 95% CI 1.01-1.05; p = 0.008).

Pre-existing CKD was associated with more than threefold higher odds of AKI (aOR 3.12, 95% CI 1.34-7.28; p = 0.008). Sepsis or septic shock was also independently associated with AKI (aOR 2.74, 95% CI 1.48-5.08; p = 0.001). Vasopressor support at ICU admission (aOR 2.86, 95% CI 1.49-5.50; p = 0.002) and mechanical ventilation at ICU admission (aOR 2.09, 95% CI 1.08-4.04; p = 0.029) were likewise independently associated with higher odds of AKI.

Higher APACHE II scores were independently associated with AKI; each one-point increase in APACHE II score was associated with a 9% increase in the odds of AKI (aOR 1.09, 95% CI 1.04-1.14; p < 0.001). Similarly, each 1 mmol/L increase in serum lactate was associated with a 31% increase in the odds of AKI (aOR 1.31, 95% CI 1.10-1.57; p = 0.003) (Table 5).

**Table 5:** Multivariable logistic regression analysis of factors independently associated with AKI Adjusted odds ratios (ORs) are presented with 95% confidence intervals (CIs). Age, APACHE II score, and lactate were analyzed as continuous variables. Mechanical ventilation and vasopressor support were defined according to status at ICU admission. Variables were entered into the multivariable model based on clinical relevance and/or univariable association with AKI. AKI: acute kidney injury; APACHE II [14]: Acute Physiology and Chronic Health Evaluation II; ICU: intensive care unit

Predictor	Adjusted OR	95% CI	p-value
Age, per 1-year increase	1.03	1.01-1.05	0.008
Sepsis/septic shock	2.74	1.48-5.08	0.001
Chronic kidney disease	3.12	1.34-7.28	0.008
Mechanical ventilation at ICU admission	2.09	1.08-4.04	0.029
Vasopressor support at ICU admission	2.86	1.49-5.50	0.002
APACHE II score, per 1-point increase	1.09	1.04-1.14	<0.001
Serum lactate, per 1 mmol/L increase	1.31	1.10-1.57	0.003

## Discussion

The aim of this retrospective study was to identify the incidence, predictors, and clinical outcomes of AKI in 261 critically ill patients in the ICU. In 102 patients (39.1%), AKI developed; a significant number progressed to moderate (34 (33.3%)) or severe (26 (25.5%)) stages with the KDIGO classification. In patients who experienced AKI, there was a significantly increased mortality rate in the ICU and during hospital stay, longer ICU and hospital stays, longer duration of mechanical ventilation, and higher rates of RRT. Multivariable analysis identified the following as independent risk factors for the development of AKI: older age, pre-existing CKD, sepsis or septic shock, mechanical ventilation, vasopressor requirement, APACHE II score, and serum lactate level on admission.

The incidence of AKI in the present study was in line with the previously reported 30%-50% of critically ill patients who developed AKI during their stay in the ICU. Variations in incidence will be expected due to patient population, severity of illness, admission criteria for intensive care, healthcare resources, and diagnostic criteria. The high percentage of patients who had KDIGO stage 2 and 3 AKI in the current study further highlights the clinical significance of AKI in critically ill patients. Previous studies have also shown that critically ill patients can go on to develop more severe stages of AKI due to the fact that renal injury is frequently due to multiple co-occurring insults such as hemodynamic instability, systemic inflammation, nephrotoxic exposure, and multiorgan dysfunction [16].

Patients who developed AKI had higher observed mortality, longer duration of mechanical ventilation, and longer ICU and hospital stays than those without AKI. ICU mortality and in-hospital mortality were significantly higher in the AKI group, while the proportion of patients discharged alive was lower. These findings are consistent with previous studies reporting an association between AKI and adverse outcomes among critically ill patients, irrespective of the underlying critical illness [17]. Renal dysfunction may contribute to adverse outcomes through fluid and electrolyte disturbances, acid-base abnormalities, impaired drug clearance, fluid overload, and progression of multiorgan dysfunction.

However, these clinical outcomes should be interpreted in the context of substantial differences in baseline and admission illness severity between the AKI and non-AKI groups. Patients who developed AKI were older and had higher frequencies of CKD and sepsis/septic shock, as well as higher APACHE II and SOFA scores and greater requirements for mechanical ventilation and vasopressor support. The comparisons of ICU mortality, in-hospital mortality, duration of mechanical ventilation, and ICU and hospital LOS were unadjusted and therefore do not establish AKI as an independent determinant of mortality or resource utilization. Although AKI was significantly associated with these adverse outcomes, residual confounding by illness severity and other measured or unmeasured factors cannot be excluded. Given the retrospective observational design, these findings should therefore be interpreted as describing the observed clinical burden associated with AKI rather than demonstrating a causal effect of AKI on mortality, mechanical ventilation duration, or LOS.

Pre-existing CKD had one of the strongest associations with AKI, with an aOR of 3.12 (95% CI 1.34-7.28, p = 0.008) among the independent predictors identified. A patient who has CKD may have less renal functional reserve and may be more susceptible to other critical illness-induced hemodynamic, inflammatory, and nephrotoxic insults. These results have been corroborated by previous studies, which have found that pre-existing CKD is one of the predisposing factors for AKI in critically ill patients [18]. These results underscore the significance of early detection of patients with CKD on admission to the ICU and careful monitoring of renal function during their ICU stay.

Sepsis or septic shock was also independently associated with AKI (aOR 2.74, 95% CI 1.48-5.08; p = 0.001). Sepsis-associated AKI is a multifactorial process, associated with systemic inflammation, endothelial dysfunction, microvascular disturbances, changes in renal perfusion, and injury. Septic AKI is a well-recognized complication of critical illness and has been related to significant morbidity and mortality, especially if combined with other multiorgan dysfunctions [19]. The close relationship revealed in the current study further highlights the need to routinely assess renal function in critically ill patients with sepsis or septic shock from an early stage.

The requirement for vasopressors and mechanical ventilation at admission to the ICU were also independently associated with AKI, with aORs of 2.86 (95% CI 1.49-5.50; p = 0.002) and 2.09 (95% CI 1.08-4.04; p = 0.029), respectively. It is important to note that these variables should mainly be understood as indicators of hemodynamic and physiological severity, and not as direct causes of AKI. The need for vasopressors is usually a measure of circulatory instability, and mechanical ventilation indicates that the patient has significant respiratory or multisystem dysfunction. These factors can be superimposed in patients with serious critical illness, along with renal hypoperfusion and systemic organ dysfunction. Mechanical ventilation and vasopressor therapy have also been shown to be significant markers of severe critical illness and risk factors for AKI in previous studies [20].

Other significant and independent risk factors for AKI were the APACHE II score and the admission serum lactate. For every 1-point increase in APACHE II score, the odds of AKI were increased by 9% (aOR 1.09, 95% CI 1.04-1.14; p < 0.001), while every 1 mmol/L increase in the lactate level at admission was associated with a 31% increase in the odds of AKI (aOR 1.31, 95% CI 1.10-1.57; p = 0.003). Increased physiological derangement and hypoperfusion at admission to the ICU were independently associated with increased risk of subsequent development of AKI, suggesting the hypothesis that these factors are more detrimental to the development of AKI. However, APACHE II and lactate should be treated as indicators of the severity of illness, not causes of kidney injury.

These findings have important clinical implications [20]. Identification of patients at higher risk of AKI based on readily available admission characteristics, including pre-existing CKD, sepsis or septic shock, elevated serum lactate, vasopressor support, mechanical ventilation, and higher APACHE II scores, may help clinicians prioritize closer monitoring of renal function and urine output, optimize hemodynamic status, review potentially nephrotoxic medications, and seek timely nephrology input where clinically indicated [21]. However, conventional KDIGO criteria based on serum creatinine and urine output may identify kidney dysfunction after substantial injury has already occurred. Identification of subclinical AKI, in which structural or tubular injury is present before conventional functional criteria are fulfilled, may therefore provide an earlier opportunity for risk stratification and intervention. Emerging biomarkers including NGAL, KIM-1, and tissue inhibitor of metalloproteinases-2 (TIMP-2)/insulin-like growth factor-binding protein (IGFBP7) have been investigated as markers of kidney stress or injury that may complement conventional functional criteria [22,23].

Future approaches may extend beyond detection toward phenotypic and subphenotypic classification of AKI. Combining clinical characteristics, trajectories of serum creatinine and urine output, biomarkers, hemodynamic variables, and underlying comorbidities may allow identification of biologically and clinically distinct AKI phenotypes or endotypes with different prognoses and potentially different responses to treatment [24,25]. Such phenotypic subtyping could support precision interventions rather than applying a uniform management strategy to all patients with AKI. In parallel, artificial intelligence and machine-learning approaches may integrate continuously updated electronic health-record data to generate dynamic risk scores that reflect changing patient status rather than relying solely on admission characteristics [26]. Real-time predictive models linked to automated electronic alerts could identify rapidly increasing AKI risk and prompt standardized clinical review, although prospective validation is required to establish whether such systems improve patient outcomes and avoid alert fatigue.

Earlier identification should ultimately be linked to actionable management. For patients at increased risk of AKI, individualized fluid and vasopressor strategies may help avoid both renal hypoperfusion and excessive fluid accumulation, with hemodynamic management guided by the underlying cause of shock and the patient's evolving volume and perfusion status. Nephrotoxic stewardship should include systematic review of potentially nephrotoxic medications, dose adjustment according to renal function, avoidance of unnecessary exposure, and careful assessment of the risk-benefit balance of contrast administration [27]. Similarly, the timing of RRT should be individualized according to clinical context rather than triggered solely by AKI stage. Evidence from randomized trials indicates that routine accelerated RRT initiation in severe AKI without urgent indications does not confer a mortality benefit, supporting careful assessment of metabolic complications, fluid status, urine output, and overall clinical trajectory when determining the need for RRT [28].

The implications of AKI also extend beyond ICU discharge. Survivors of critical-illness-associated AKI may remain at increased risk of persistent renal dysfunction, CKD, recurrent AKI, and cardiovascular complications [29]. Post-ICU care should therefore include reassessment of renal function and albuminuria where appropriate, blood-pressure and diabetes management, medication reconciliation and renal dose adjustment, avoidance of unnecessary nephrotoxic exposure, and nephrology follow-up for patients with persistent renal dysfunction or severe AKI, particularly those who required RRT. Integration of these measures into post-ICU survivorship pathways could facilitate earlier recognition of incomplete renal recovery and allow comorbidity management to be adapted according to the patient's evolving kidney function.

Taken together, the future management of critically ill patients at risk of AKI may increasingly involve a continuum from early biomarker-based detection and phenotypic classification to dynamic risk prediction, automated clinical decision support, individualized hemodynamic and nephrotoxic stewardship, appropriately timed RRT, and structured post-ICU renal surveillance. Nevertheless, these strategies require prospective validation, cost-effectiveness assessment, and evaluation in diverse healthcare settings before routine implementation, particularly in resource-constrained environments.

This study is limited in several ways that need to be taken into consideration when examining this study's findings. First, its single-center, retrospective design does not allow it to draw conclusions about cause and effect, and may make the results less applicable to other centers with different patient populations, disease patterns, and critical care practices. Second, this sample size was relatively small (261 patients), which may have reduced the power to detect smaller associations and to allow meaningful subgroup analyses. Third, information bias may have occurred because certain clinical parameters may not have been well documented or complete, despite attempts to include patients with appropriate, complete, and relevant clinical information.

Fourth, detailed information regarding several potentially important determinants of AKI was not consistently available for analysis, including cumulative fluid balance, dose and duration of nephrotoxic medications, timing of antibiotic exposure, longitudinal urine output, and comprehensive hemodynamic measurements. As a result, it is impossible to rule out residual confounding caused by factors that were not measured or were only partially measured. Fifth, the study did not evaluate long-term renal outcomes, such as renal recovery, persistent kidney dysfunction, or progression to CKD after discharge, but instead focused on AKI during the ICU and hospital course. Finally, external validation in prospectively larger multicenter populations is required for the predictive associations found in this single-center cohort. To validate these predictors, better define the temporal relationship between potentially modifiable exposures and AKI, and determine their relationship with long-term renal and patient outcomes, additional multicenter prospective studies with larger sample sizes and longer follow-up periods are required.

## Conclusions

In this study, AKI was associated with a greater observed clinical burden among critically ill patients, including higher requirements for RRT, longer ICU and hospital stays, prolonged mechanical ventilation, and higher ICU and in-hospital mortality. Pre-existing CKD, sepsis or septic shock, vasopressor support at ICU admission, mechanical ventilation at ICU admission, higher serum lactate, higher APACHE II score, and increasing age were independently associated with the development of AKI. Because the clinical outcome comparisons were unadjusted, the observed differences in mortality, mechanical ventilation duration, and LOS do not establish AKI as an independent determinant of these outcomes. Early identification of patients at increased risk, close monitoring of renal function and urine output, optimization of hemodynamic status, and appropriate review of potentially nephrotoxic exposures may support timely recognition and management of AKI in critically ill patients.

## Appendices

Data Collection Form

Study title: Incidence, Predictors, and Clinical Outcomes of Acute Kidney Injury in Critically Ill Patients: A Retrospective Study

A. Patient Identification and Eligibility

Study identification number: __________

Age: ______ years

Sex: Male/Female

ICU admission date: __________

ICU discharge date: __________

ICU stay ≥ 24 hours: Yes/No

Inclusion criteria fulfilled: Yes/No

Exclusion criteria present: Yes/No

Reason for exclusion, if applicable: __________

B. Demographic and Baseline Characteristics

Age: ______ years

Sex: Male/Female

BMI: ______ kg/m²

Smoking history: Yes/No

Diabetes mellitus: Yes/No

Hypertension: Yes/No

Chronic kidney disease: Yes/No

Ischemic heart disease: Yes/No

Chronic liver disease: Yes/No

Malignancy: Yes/No

C. ICU Admission Characteristics

Primary ICU admission diagnosis: __________

Sepsis/septic shock: Yes/No

Mean arterial pressure: ______ mmHg

Vasopressor requirement at ICU admission: Yes/No

Mechanical ventilation at ICU admission: Yes/No

APACHE II score: ______

SOFA score: ______

D. Admission Laboratory Parameters

Serum creatinine: ______ mg/dL

Blood urea nitrogen: ______ mg/dL

Serum lactate: ______ mmol/L

Hemoglobin: ______ g/dL

WBC count: ______ ×10⁹/L

Serum albumin: ______ g/dL

Sodium: ______ mmol/L

Potassium: ______ mmol/L

Arterial pH: ______

Other relevant laboratory parameters: __________

E. AKI Assessment

AKI developed: Yes/No

Date/time of first AKI documentation: __________

KDIGO stage:

Stage 1

Stage 2

Stage 3

Maximum serum creatinine: ______ mg/dL

Urine output criterion fulfilled: Yes/No

Date/time of AKI onset: __________

F. Pre-AKI Clinical Exposures

Nephrotoxic medication exposure before AKI: Yes/No

Iodinated contrast exposure before AKI: Yes/No

Surgical procedure before AKI: Yes/No

Blood transfusion before AKI: Yes/No

Other relevant exposure: __________

G. Clinical Outcomes

Renal replacement therapy: Yes/No

Duration of mechanical ventilation: ______ days

ICU length of stay: ______ days

Hospital length of stay: ______ days

ICU mortality: Yes/No

In-hospital mortality: Yes/No

Discharged alive: Yes/No
